# Venous Ligation: A Novel Strategy for Glans Enhancement in Penile Prosthesis Implantation

**DOI:** 10.1155/2014/923171

**Published:** 2014-08-07

**Authors:** Geng-Long Hsu, James W. Hill, Cheng-Hsing Hsieh, Shih-Ping Liu, Chih-Yuan Hsu

**Affiliations:** ^1^Microsurgical Potency Reconstruction and Research Center, Hsu's Andrology and National Taiwan University, 3F 88, Wen-Hu Street, Neihu District, Taipei City 11445, Taiwan; ^2^Department of Urology, National Taiwan University Hospital, College of Medicine, Taipei City 10048, Taiwan; ^3^Department of Radiology, University of Southern California, Los Angeles, CA 90033, USA; ^4^Division of Urology, Buddhist Tzu-Chi General Hospital, Taipei Branch, School of Medicine, Buddhist Tzu-Chi University, Hualien City 970, Taiwan

## Abstract

Although penile implantation remains a final solution for patients with refractory impotence, undesirable postoperative effects, including penile size reduction and cold sensation of the glans penis, remain problematic. We report results of a surgical method designed to avoid these problems. From 2003 to 2013, 35 consecutive patients received a malleable penile implant. Of these, 15 men (the enhancing group) were also treated with venous ligation of the retrocoronal venous plexus, deep dorsal vein, and cavernosal veins. The remaining 20 men formed the control group, treated with only a penile implant. Follow-up ranged from 1.1 to 10.0 years, with an average of 6.7 ± 1.5 years. Although preoperative glanular dimension did not differ significantly between the two groups, significant respective difference at one day and one year postoperatively was found in the glanular circumference (128.8 ± 6.8 mm versus 115.3 ± 7.2 mm and 130.6 ± 7.2 mm versus 100.5 ± 7.3 mm; both *P<0.05*), radius (38.8 ± 2.7 mm versus 37.1 ± 2.8 mm and 41.5 ± 2.6 mm versus 33.8 ± 2.9 mm; latter *P<0.01*), and satisfaction rate (91.7% versus 53.3%, *P<0.01*) as well. Based on our results, selective venous ligation appears to enhance the glans penis dimension in implant patients.

## 1. Introduction

The human penis has been in its current anatomical form for a couple of thousand centuries [1]. In our comparative study of penile anatomy in quadruped and biped animals [2], the former consistently possess an os penis that is virtually free from rigidity problems, whereas humans are peculiar among bipedal animals in possessing disproportionately large and extraordinarily hydraulic corpora cavernosa (CC), an os analog, which prevents the glans from being too feeble for intromission. Interestingly, the glans makes no contribution to the necessary rigidity of the penile shaft [3]. The erectile capability of the human penis largely depends on sinusoids in the glans penis, the corpus spongiosum, and the CC, the latter of which are also exclusively responsible for overall erection rigidity [4, 5]. The human penis frequently encounters erectile dysfunction (ED), defined as inability either to attain or to maintain rigid erection for satisfactory intercourse [6].

Although we have lived in the era of medical treatment of ED since sildenafil was introduced in 1998 [7], penile implantation remains the final viable solution for many patients with refractory ED. The overall number of penile implantations per year rebounded after a temporary dip following introduction of sildenafil [8]. Penile prosthesis has been the best option to provide reliable penile rigidity in many ED patients [9, 10], and it may be performed under local anesthesia [11–14]. Nevertheless, many candidates are reluctant to accept this treatment, because it is not natural and some adverse outcomes can occur, such as prosthesis loss, sinusoidal damage, a need for revision surgery, and seemingly intolerable postoperative consequences such as a cold, smaller, and wrinkled glans penis, shortening of the penile shaft, and even loss of penile perception. Among these, glanular problems stand out. Herein we found that venous ligations at a retrocoronal level constitute a viable option for reducing the incidence of glanular size reduction. The techniques outlined herein were refined over the course of extensive clinical practice and cadaveric studies of penile tunical and venous anatomy [15–17].

## 2. Materials and Methods

**Figure 1:**
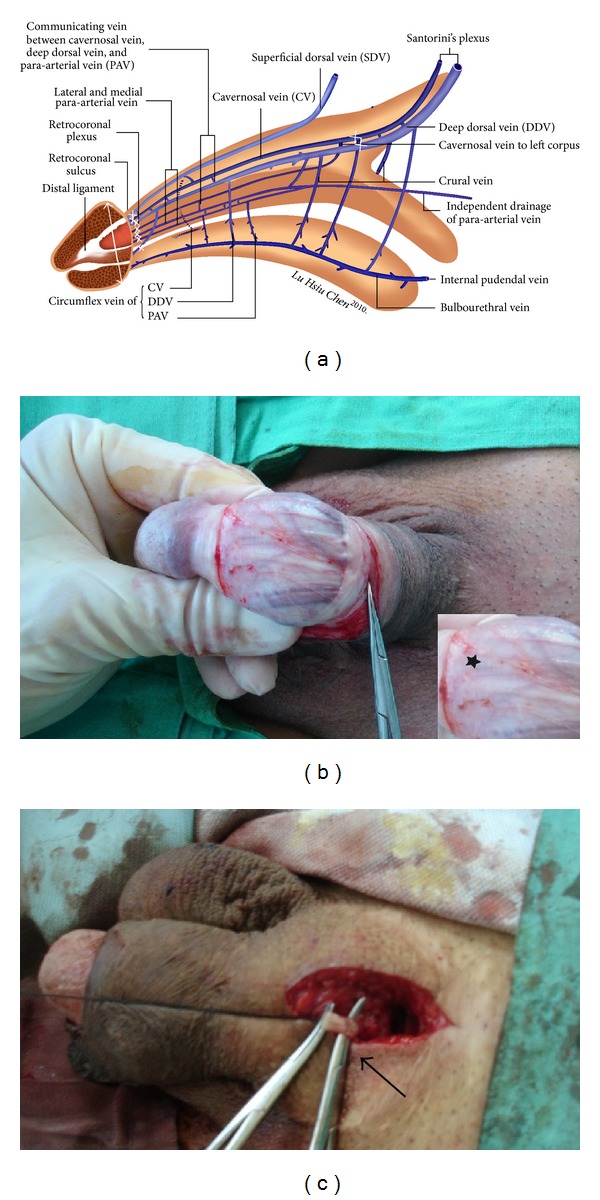
Schematic illustration and photos of this penile enhancing surgery. (a) Illustration showing new insight into penile venous anatomy from lateral view in the human penis. The glans penis composed of sinusoids through which blood drains independently to the deep dorsal vein (DDV), cavernosal veins (CVs), and para-arterial veins. The venous plexus were ligated at retrocoronal sulcus (multiple smaller cross). DDV and CVs were subsequently ligated close to penile hilum (large cross). The radius of glans was assessed (double arrow). (b) Ongoing surgery demonstrating the visibility of the retrocoronal plexus (asterisk) can be enhanced via squeezing the glanular sinusoids after a circumferential approach was performed. Segment of 1-2 cm was stripped while the ligation number may be as many as 29. (c) The proximal segment of DDV (clamped by mosquito hemostat, arrow) and CVs was freed and ligated close to penile hilum.

From 2003 to 2013, a total of 35 ED patients, aged from 37 to 75 years, received a single-piece penile implant with either malleable or mechanical prosthesis under an acupuncture-aided local anesthesia on an outpatient basis. Penile dimension was obtained in terms of glanular circumference and radius measured along the corona of the glans penis (Figure 1(a)), while the penile stretch length was recorded and then glandular radius was reassessed on 30-degree oblique pelvic X-ray film. Of these, 15 men, each of whom expressed concern about a loss of postoperative penile dimension, were allocated into an enhancing group and were treated with venous ligation of the retrocoronal venous plexus (Figure 1(b)) and proximal ligation of the deep dorsal vein and cavernosal veins (Figure 1(c)) in addition to regular penile implantation. The remaining 20 males were treated with just standard penile implantation and were regarded as a control group. In the enhancing and control group the types of prosthesis used were 4, 2, 4, 2, and 3 versus 4, 3, 6, 4, and 3 to AMS Spectra, Mentor Acuform, AMS600, AMS650, and Dacromed Duraphase II, respectively.

### 2.1. Venous Ligation and Penile Implant

**Figure 2:**
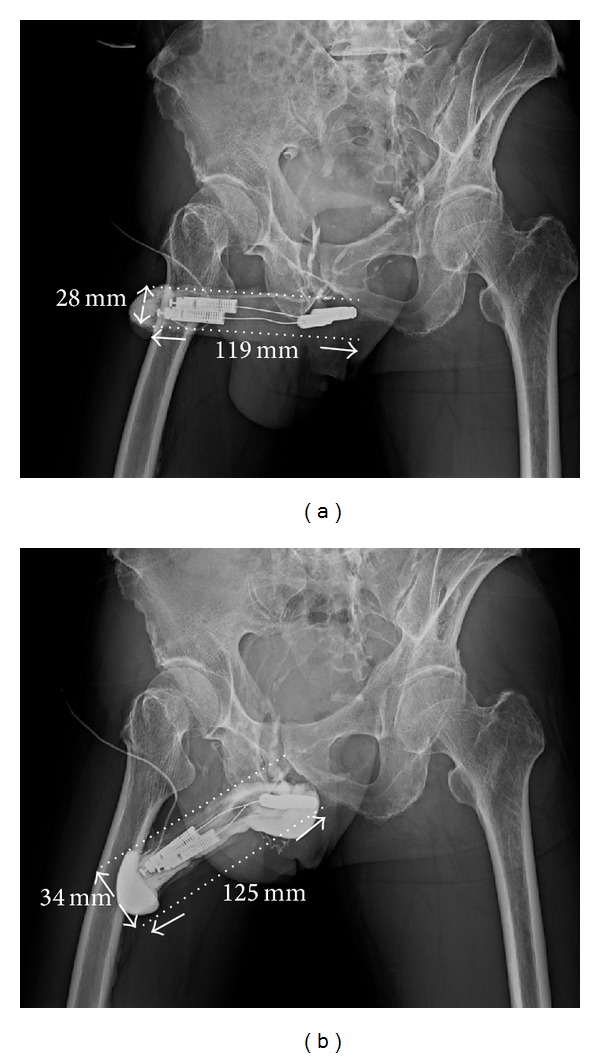
Pelvic X-ray film of 30° oblique view of a 65-year-old male. He underwent the first surgery somewhere in 2005. A cold glans syndrome prompted to receive the venous ligation surgery. (a) The glanular radius was enhanced from 28 mm to 34 mm after the penile venous surgery. The corporeal length was 119 mm from X-ray, and it was 180.0 mm from implant surgery; however 90.0 mm × tan⁡ 60° (1.73205080757) = 206.1 mm. (b) The DDV was ligated at the level of retrocoronal and hilum region. Enhancement was demonstrated in both the glans penis and entire penile shaft after a contract medium was injected to the glans penis via a #23 scalp needle.

**Figure 3:**
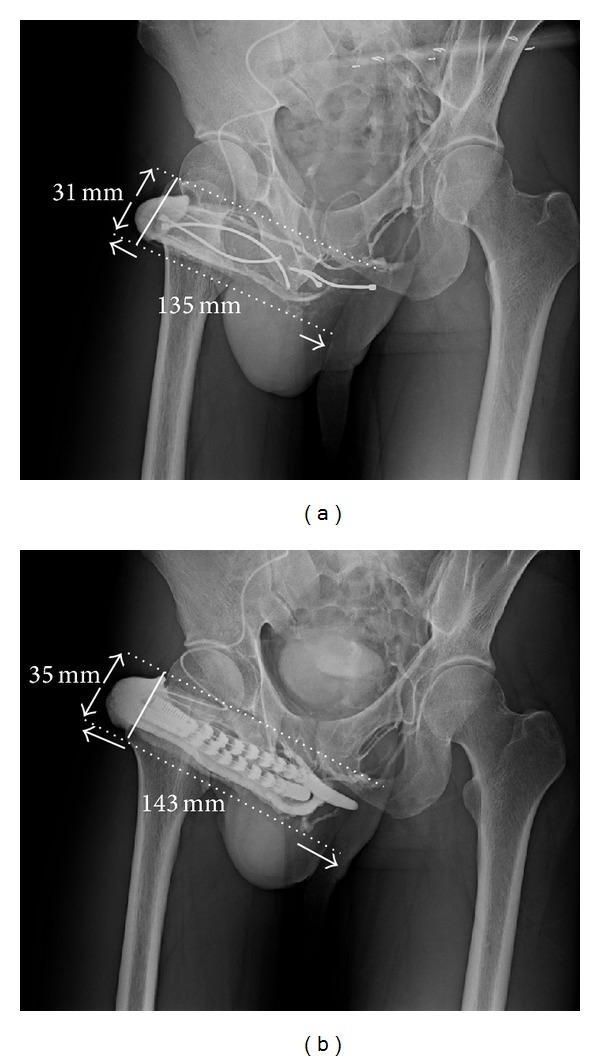
Pelvic X-ray film of 30° oblique view of a 35-year-old male of traumatic impotence. He underwent the first surgery somewhere in 2006. A mechanical failure of penile prosthesis prompted him to receive an implant revision and the venous ligation surgery for cold glans syndrome. (a) The glanular radius was enhanced from 31 mm to 35 mm after the penile venous surgery. (b) The DDV was ligated at the level of retrocoronal and hilum region. Enhancement was shown in both the glans penis and entire penile shaft after a contract medium was injected to the glans penis via a #23 scalp needle.

**Figure 4:**
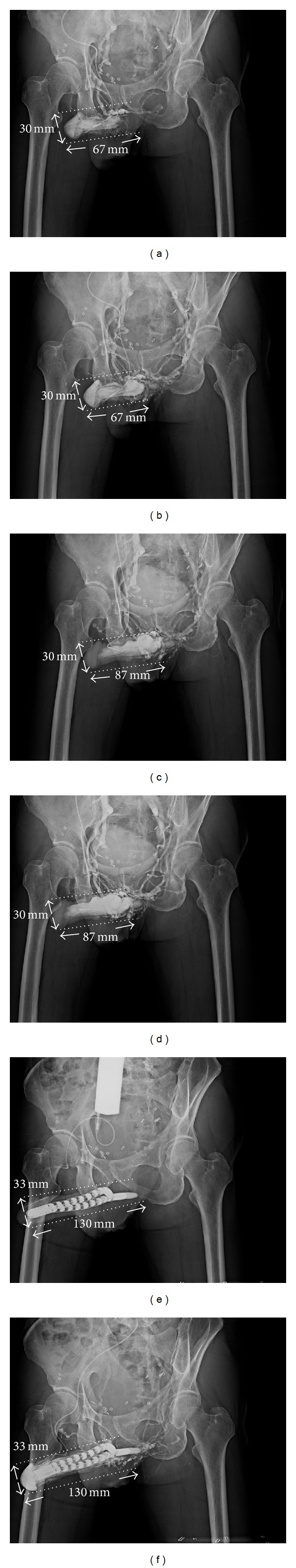
Pelvic X-ray film of 30° oblique view of a 77-year-old male of traumatic impotence. He underwent cryosurgery for prostate adenocarcinoma in 2010. (a) Cavernosogram was made after 20 mL of contract medium was injected. (b) Cavernosogram was undertaken after another 30 mL of contract medium was injected. (c) The penile tissue could not extend 30 min after 20 *μ*g prostaglandin E1 (PGE1) was intracavernously injected. The venous leakage was shown because the drainage veins are conspicuous despite an intracavernosal pressure which exceeded 110 mmHg. (d) The situation was reassured. (e) The venous surgery was performed for penile enhancement in addition to regular penile implant. The penile length was increased although the glandular radius changed from 30 mm to 33 mm. This situation is confirmed (f).

These procedures were initiated with acupuncture-aided local anesthesia [18]. The operative time was recorded from the time of injecting the local anesthetic to the completion of skin suturing. A circumferential subcoronal incision was standard for regular penile implantation in all patients [19]. Thus the implantation was made following a 4 cm corporotomy which was performed on the distal-lateral corpus bilaterally. The tunical wound was sutured with 6-0 nylon continuously with exact approximation of the tunica albuginea and subsequently with interrupted sutures at each 1.5 cm interval for enhancement. The overlying fascia layers and skin were approximated with 5-0 chromic suture, layer by layer. In the enhancing group before penile implantation was performed, a meticulous venous dissection was made along the dorsal retrocoronal region, based on new insights of penile venous anatomy (Figure 1(a)). The visibility of drainage veins of the glans penis could be enhanced via manual squeezing on the glans (Figure 1(b)). They were meticulously stripped for at least a 1.0 cm segment and then ligated with 6-0 nylon sutures, resulting in 29 ligatures in total. Proximally venous ligations were made on the deep dorsal vein and cavernosal vein (Figure 1(c)) deep to the penile hilum as much as possible. The glans radius was reassessed on postoperative X-ray (Figures 2, 3, and 4). Corporeal length and glandular dimension were also analyzed manually. These were followed annually. Overall satisfaction rate was also recorded in both groups. Statistical Mann-Whitney U and Fisher's exact test were applied where appropriate.

## 3. Results

The follow-up time was from 1.1 to 10.0 years with an average of 6.7 ± 1.5 years. Loss of follow-up occurred in 3 and 5 men in the enhancing and control group, respectively. Among them, 2 and 4 males died. To provide a comprehensive overview, Table 1 summarizes demographic data of the 35 patients. The operative time was 45.0–67.0 min (average 52.3 ± 5.5 min) and 101.5−117.8 min (average 121.7 ± 6.8 min) for the control and enhancing group, respectively. There was no difference in the preoperative glanular circumference between groups (112.7 ± 15.8 mm versus 113.6 ± 13.2 mm; *P* = 0.55). Although the operation time was significantly protracted (121.7 ± 6.8 min versus 52.3 ± 5.5 min; *P* < 0.001), there was a significant difference between the enhancing and control groups at one day and one year postoperatively in glanular circumference (128.8 ± 6.8 mm versus 115.3 ± 7.2 mm and 130.6 ± 7.2 mm versus 100.5 ± 7.3 mm, resp.; both *P* < 0.05) and glanular radius (38.8 ± 2.7 mm versus 37.1 ± 2.8 mm and 41.5 ± 2.6 mm versus 33.8 ± 2.9 mm, resp.; latter *P* < 0.01).

Postoperative satisfaction rate was greater in the enhancing group (91.7% versus 53.3%, *P* < 0.01). In the control group, 45% (9/20) of patients complained of a cold glans. No patients in the enhancing group reported this problem. Corporeal length was 18.2 cm and 18.1 cm in manual measurement in the enhancing and control group, respectively, and its corresponding measurement was 13.5 and 13.6 cm, respectively, on 30-degree oblique film.

## 4. Discussion

Where rigidity is concerned, humans have not benefitted from penile evolution, advancing from the os penis (a rigid body) in quadrupeds to the CC (a hydraulic system) in upright animals [20]. Not surprisingly, pursuits for penile rigidity appear endlessly in human history. The development of the penile implant is being a good example [21]. An implanted penis may mitigate rigidity problems but unfortunately may place the penis at risk not only of compromising tissue integrity [22], but also of penile dimension reduction once the CC are implanted. Several studies support these concerns [12, 23]. We acknowledge the variability of manual measurements of penile dimension, which lack a universal standard. In this series, we use objective criteria based on a 30° oblique X-ray film. Those data were corrected by tangent 60° (tan⁡ 60° = 1.73205080757), and smaller values were still demonstrated in each corresponding parameter. Thus, parameters from X-ray film may be difficult to compare with those from manual measurement (Table 1 182.3 ± 8.2 and 181.5 ± 8.4 to enhancing and control group, resp.) and that by X-ray (135.3 ± 7.9 and 136.3 ± 8.5 correspondingly) because discrepancy exists consistently. However, evaluating penile dimension is reliable if comparison is made based on chronological X-ray films.

Although extensive studies of human penis have been performed, an understanding of its anatomy may leave room for improvement [24]. The sinusoids of the corpora cavernosa (CC) differ from those in the corpus spongiosum (CS), which is capped with the glans penis, containing the same sort of sinusoids. Are there, therefore, only two types of sinusoids in the human penis? In our study, the CC, CS, and glans penis each possess specific types of sinusoids histologically [25]. It was accordingly hypothesized that blockage of the draining veins of glanular sinusoids might encourage gradual growth of glanular volume [26]. The venous ligation technique presented here confirms this in our experience [27, 28].

The loss of penile length and the appearance of glans coldness after implantation appear unavoidable in some cases, and several studies have aimed to solve these problems [29–33]. Fortunately, many patients might not care much once their rigidity is improved. However, these problems are bothersome for some men. In this series, three males underwent a first penile implantation somewhere else and requested a viable solution for cold glans syndrome. This problem was mitigated by penile implant revision and the glanular enhancement procedure described herein, resulting in satisfactory outcomes (Figures 2 and 3). Applying this novel method of penile enhancement could benefit cold glans syndrome in patients with penile implant. Further, an acupuncture-aided pure local anesthesia has permitted patients to return to casual activity promptly with negligible morbidity [34].

## 5. Conclusion

In conclusion, a combination of venous stripping of the retrocoronal plexus and ligation of the DDV and CVs at the penile hilum appears to enhance glanular dimension in implant patients and may treat cold glans syndrome. Studies of larger numbers of patients are required.

## Figures and Tables

**Table 1:** Summary of 35 implant patients who underwent venous ligation for penile enhancement in implant patients.

Grouping	Patients		Circumference of glans corona Manual measurement (mm)			Radius of glans penis Manual measurement (mm)			Corporal length Surgery (mm)	Corporeal length X-ray (mm)	Satisfaction rate Number/available (%)
	Number	Age	Preop	Postop (1 day)	Postop (1 year)	Preop	Postop (1 day)	Postop (1 year)			
Enhancing	15	37–75	112.7 ± 15.8	128.8 ± 6.8	130.6 ± 7.2	37.3 ± 2.9	38.8 ± 2.7	41.5 ± 2.6	182.3 ± 8.2	135.3 ± 7.9	11/12 (91.7)
Control	20	41–75	113.6 ± 13.2	115.3 ± 7.2	100.5 ± 7.3	36.9 ± 2.4	37.1 ± 2.8	33.8 ± 2.9	181.5 ± 8.4	136.3 ± 8.5	8/15 (53.3)
Total	**35**										

*P* value^†^		NS∗	0.55	<0.05	<0.01	NS∗	NS∗	<0.01	NS∗	NS∗	<0.01

*NS stands for not significant with *P* value of greater than 0.05.

^†^Univariate comparisons were performed using the Mann-Whitney *U* test as necessary for parameters with continuous values and Fisher's exact test with discontinuous parameters.
